# Supplementary material for: Frequency, Treatment and Outcome of Immune-Related Toxicities in Patients with Immune-Checkpoint Inhibitors for Advanced Melanoma: Results from an Institutional Database Analysis
Source: Cancers (Basel). 2021 Jun 11;13(12):2931. doi: 10.3390/cancers13122931 (PMC8230729; doi:10.3390/cancers13122931)
Supplement: Supplementary file 1 [file cancers-13-02931-s001.zip › cancers-1237050-supplementary.pdf]

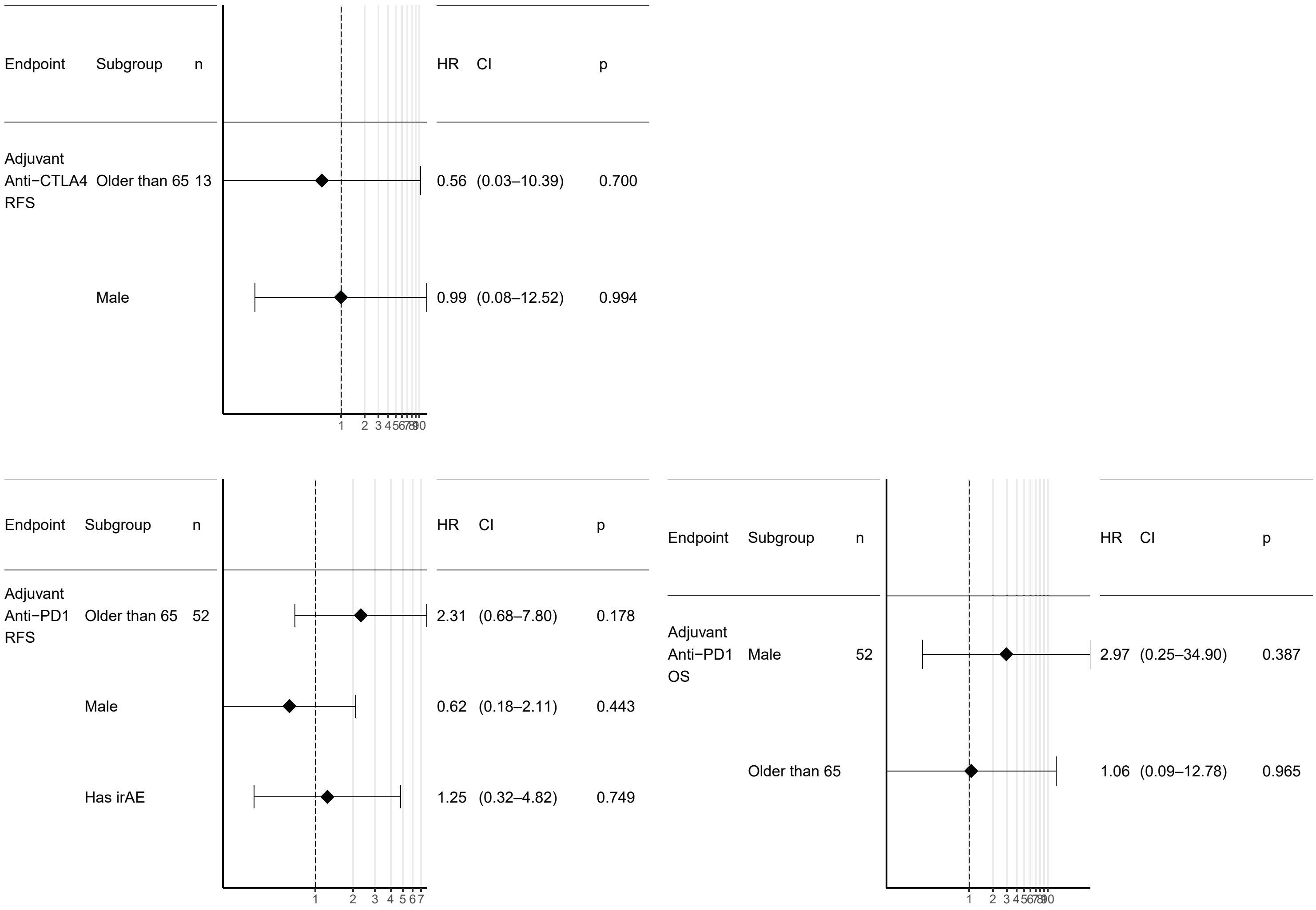

Figure S1: Univariate Cox regression analysis for covariates in the adjuvant setting.

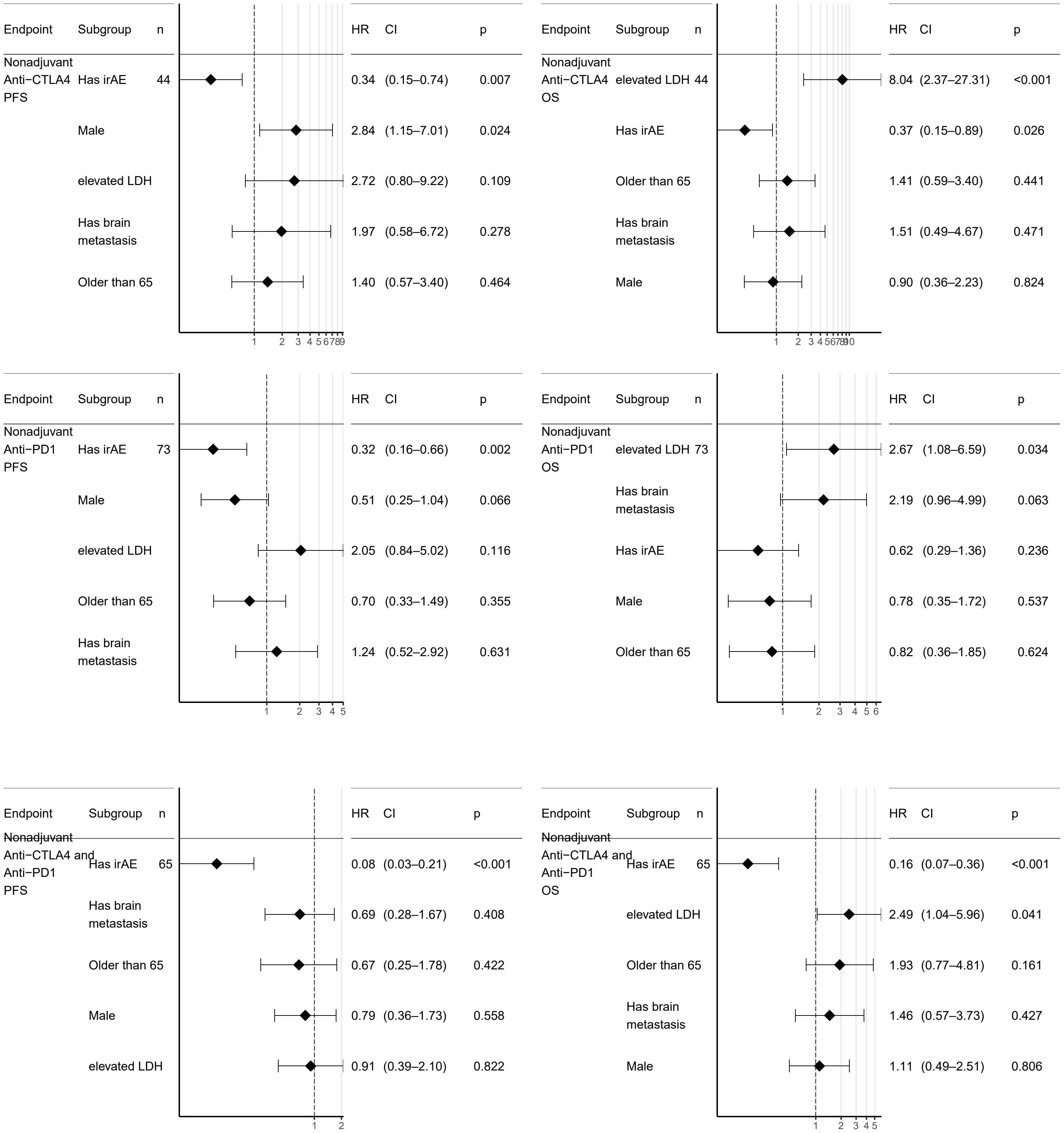

Figure S2: Univariate Cox regression analysis for covariates in the unresectable/non-adjuvant setting.
